# Editorial: Resilience, quality of life and psychosocial outcomes of cancer patients and their caregivers

**DOI:** 10.3389/fpsyg.2023.1324977

**Published:** 2023-11-13

**Authors:** Nida Zahid, Nargis Asad, Ashraf El-Metwally

**Affiliations:** ^1^Department of Surgery, Aga Khan University Hospital, Karachi, Pakistan; ^2^Department of Women's and Children's Health, Uppsala University, Uppsala, Sweden; ^3^Department of Psychiatry, Aga Khan University, Karachi, Pakistan; ^4^Department of Public Health, King Saud bin Abdulaziz University for Health Sciences, Riyadh, Saudi Arabia; ^5^King Abdullah International Medical Research Center, Riyadh, Saudi Arabia

**Keywords:** cancer, quality of life, psycho-oncology, depression, anxiety, social support, caregivers, resilience

Worldwide, cancer is the second most common cause of death (WHO, 2018). Cancer patients suffer clinically significant symptoms of emotional distress, including depression and anxiety (Singer et al., 2010) which, in turn, detrimentally affect their quality of life (QoL) and resilience, while also posing challenges to their adherence to treatment (Weisman, 1979; Kroenke et al., 2010). Studies have revealed that individuals with similar diseases and treatment statuses experience marked disparities in their quality of life (Epping-Jordan et al., 1999; Lawford and Eiser, 2001).

Our Research Topic encompassed several themes, including resilience, quality of life, anxiety, and depression in both cancer patients and their caregivers. The selected articles for our study revolved around quality of life, suicidal ideation, depression, anxiety, as well as interventions aimed at alleviating depression and anxiety. Of the articles included, five were observational analytical studies, one was a brief report, and two were experimental studies.

In a cross-sectional study led by Ayub et al., the primary aim was to assess the factors that impact the quality of life (QoL) in breast cancer patients in Pakistan. The study's results revealed that factors such as age, entitlement, recurrence, marital status, income, the number of treatment doses, duration of cancer therapy, and the frequency of chemotherapy sessions had a significant association with QoL. A cross-sectional study carried out in China by Shen et al., the primary objective was to investigate the correlation between social constraints and the quality of life experienced by hematopoietic stem cell transplantation (HCT) survivors. Furthermore, their secondary goal was to elucidate the chain mediating role of illness perceptions and the fear of cancer recurrence in this connection. The study findings supported the conclusion that social constraints exert a detrimental impact on the quality of life of HCT survivors, with illness perceptions and the fear of cancer recurrence acting as mediating factors within this intricate relationship. In a study carried out by Li J. et al. from China, the primary objective was to investigate the intimate connection between pancreatic cancer patients and their spouse caregivers, focusing on how they provide support while dealing with anxiety and depression. This study illustrated the deep interconnection between pancreatic cancer patients and their caregiving spouses when it comes to coping with the disease. It underscored the importance of maintaining a strong, healthy intimate relationship and effective dyadic coping strategies in order to ease the burden of the illness and reduce the psychological strain experienced by families dealing with cancer. Chen J. et al. undertook a cross-sectional study in China with the objective of investigating whether depression acts as a mediator in the connection between symptom distress and suicidal ideation among ovarian cancer females. The findings from this study strongly imply that depression serves as the underlying mechanism that links symptom distress to suicidal ideation. Furthermore, the research highlights the potential of suicide resilience as a protective factor that can mitigate this risk. These findings underscore the importance of conducting future intervention research focused on enhancing suicide resilience in clinical practice. This approach aims to empower individuals to better manage their mental health challenges and life adversities, ultimately reducing the likelihood of suicidal behavior.

A brief report by Tufo et al. from Italy focused on patients' health perception 15 years post-surgery within the broader framework of overall patient wellbeing. This study emphasized the critical importance of preserving patients' functional abilities and taking a comprehensive approach to evaluate patients' wellbeing. It underscored the need to address not only disease-specific symptoms but also more general aspects of QoL to ensure a holistic approach to patient care.

Chen Y.-p. et al. from China conducted a comparative study examining the impact of different surgical approaches, including robot-assisted thoracic surgery (RATS), video-assisted thoracic surgery (VATS), and thoracotomy, on the psychological wellbeing, medical coping strategies, and overall quality of life among lung cancer patients. The study findings revealed that depression levels increased and the quality of life decreased after surgery, irrespective of the specific surgical approach employed. Notably, no significant differences were observed in these outcomes based on the surgical approach. Consequently, this study offers valuable insights, serving as a point of reference and a theoretical foundation for healthcare professionals, emphasizing the need for timely attention and intervention strategies to address the psychological and quality of life changes experienced by lung cancer patients post-surgery.

There were two experimental studies. A study conducted by Lemoine et al. was a randomized controlled trial that aimed to evaluate the effectiveness of conversational hypnosis (CH) in alleviating anxiety among breast cancer patients in France who were undergoing preoperative wire placement guided by radiographic imaging. The findings of this research did not reveal any significant advantages associated with the use of conversational hypnosis in reducing anxiety levels among patients scheduled for breast cancer surgery marker placement. Another experimental study by Li X. et al. from China analyzed the application effect of pelvic floor rehabilitation exercise in postoperative patients with cervical cancer and the factors influencing their self-efficacy. Implementing pelvic floor rehabilitation exercise for postoperative patients with cervical cancer can speed up the recovery of pelvic organ function and reduce the occurrence of postoperative urinary retention.

In summary, addressing mental health in cancer patients is of paramount significance, as it positively impacts the patient's wellbeing, treatment outcomes, and survivorship, as well as reducing the burden on caregivers and minimizing social and economic challenges. It plays a crucial role in comprehensive cancer care and contributes to the overall health and quality of life of individuals facing a cancer diagnosis.

## Author contributions

NZ: Conceptualization, Project administration, Supervision, Writing – original draft, Writing – review & editing. NA: Conceptualization, Supervision, Writing – review & editing. AE-M: Conceptualization, Supervision, Writing – review & editing.
